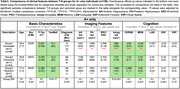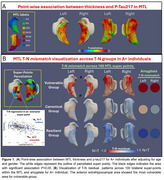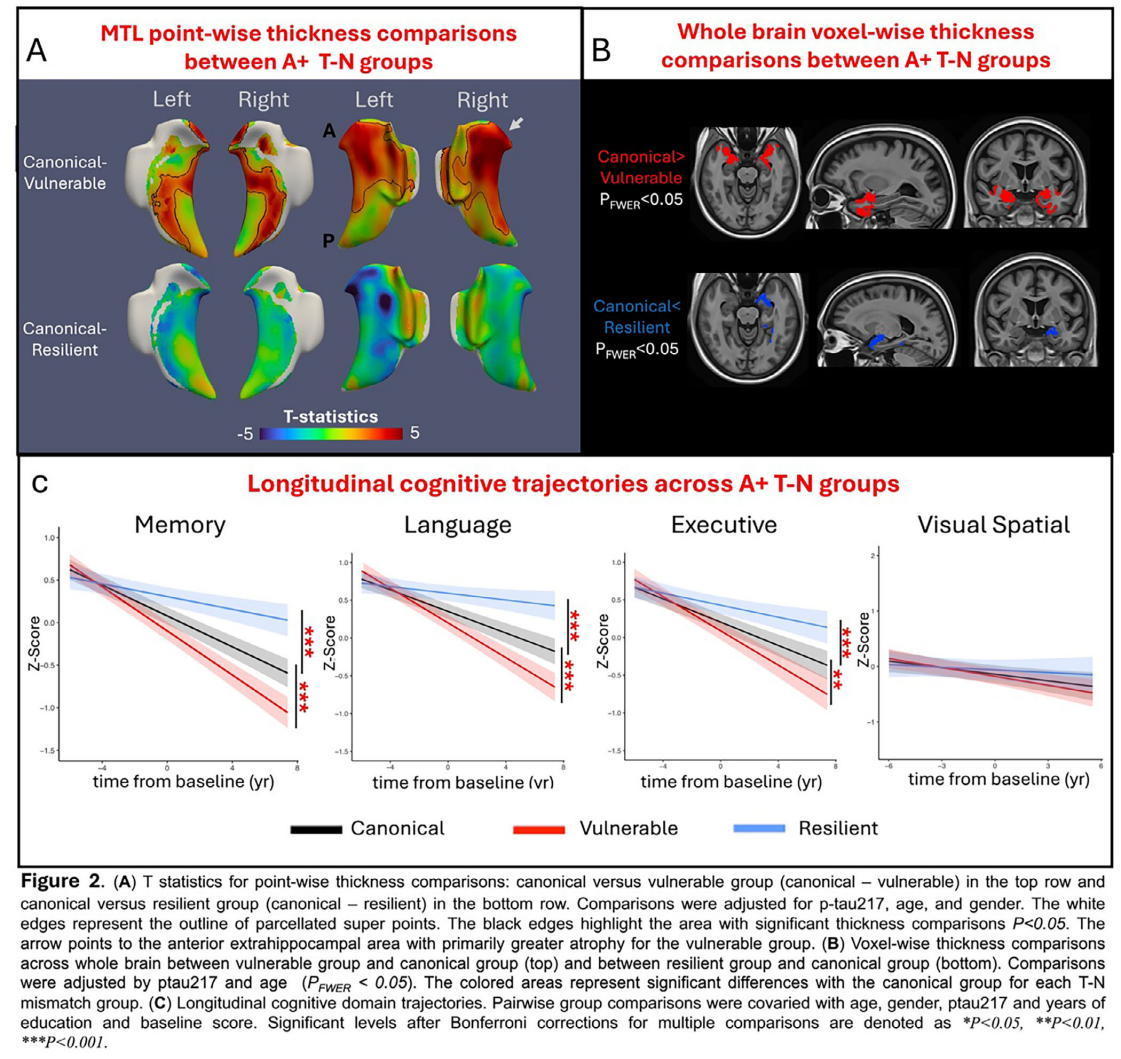# Medial temporal lobe Tau‐Neurodegeneration *mismatch* from MRI and plasma biomarkers identifies vulnerable and resilient phenotypes with AD

**DOI:** 10.1002/alz70856_107051

**Published:** 2026-01-10

**Authors:** Xueying Lyu, Nidhi S. Mundada, Christopher A Brown, Niyousha Sadeghpour, Emily McGrew, Michael Tran Duong, Long Xie, Mengjin Dong, Yue Li, Ilya M. Nasrallah, Laura E.M. Wisse, Paul A. Yushkevich, Sandhitsu R. Das, David A. Wolk

**Affiliations:** ^1^ University of Pennsylvania, Philadelphia, PA, USA; ^2^ Diagnostic Radiology, Department of Clinical Sciences Lund, Lund University, Lund, Sweden; ^3^ Penn Image Computing and Science Laboratory (PICSL), University of Pennsylvania, Philadelphia, PA, USA; ^4^ Department of Neurology, Perelman School of Medicine, University of Pennsylvania, Philadelphia, PA, USA

## Abstract

**Background:**

The heterogeneity of Alzheimer's disease (AD) and lack of well‐validated markers of non‐AD factors (e.g. TDP‐43) present a substantial challenge for therapeutics. Our prior work showed discordance between tau (T) and neurodegeneration (N) identified non‐AD factors in AD through multi‐modality imaging. Here we tried a simplified approach using plasma ptau217 and medial temporal lobe (MTL) morphometry, given this region's common association with co‐pathologies, particularly LATE‐NC.

**Method:**

We included 349 ADNI participants (188 cognitively normal, 161 MCI/dementia) with paired T1‐MRI and plasma ptau217. The MTL was segmented into subregions and further parcellated into 100 bilateral super‐points within regional boundaries. T1‐MRI‐derived thickness and amygdala volume represented N, and plasma *p*‐Tau217 represented T. T‐N residuals, calculated through regression across super‐points and amygdala, were used for weighted clustering.

**Result:**

P‐Tau217 showed strong association with MTL atrophy (Figure 1A). Three distinct data‐driven T‐N groups were identified based on mismatch patterns (Figure 1B), including a canonical group (N∼T), a vulnerable group (N>T) with negative residuals primarily in anterior hippocampal and extrahippocampal areas, and a resilient group (N<T) with positive residuals.

After clustering, group comparisons were restricted to the AD continuum (i.e. A+). While groups differed in regional volumes (e.g., amygdala), tau severity did not vary (Table 1), suggesting these patterns were not driven by AD pathology. The vulnerable group, displayed greater anterior MTL atrophy aligning with their T‐N residual patterns, while the resilient group had less atrophy in anterior extrahippocampal area (Figure 2A). Outside the MTL, the vulnerable group showed greater anterior limbic atrophy whereas the resilient group showed less (Figure 2B).

The T‐N groups differed in Clinical Dementia Rating (CDR) with the vulnerable group having the worst ratings and the resilient group the best (Table 1). Notably, the vulnerable group demonstrated greater baseline memory impairment. Longitudinally, the vulnerable group also declined more severely across multiple cognitive domains while resilient group remained most stable (Figure 2C).

**Conclusion:**

T‐N *mismatch* within MTL using MRI and plasma biomarkers revealed groups with varying vulnerability/resilience, with the vulnerable group displaying patterns of atrophy and cognition suggestive of LATE‐NC. It offers a less invasive, cost‐effective method for stratifying individuals for therapeutic interventions.